# ONLAY VERSUS SUBLAY TECHNIQUES FOR INCISIONAL HERNIA REPAIR: 30-DAY POSTOPERATIVE OUTCOMES

**DOI:** 10.1590/0102-672020220002e1692

**Published:** 2022-11-14

**Authors:** Eduardo Ferreira Martins, Marcos Dal Vesco, Pedro Klanovichs Martins, Lucas Dos Santos Difante, Lara Luz de Miranda Silva, Henrique Rasia Bosi, Bernardo Silveira Volkweis, Leandro Totti Cavazzola

**Affiliations:** 1 University Hospital of Porto Alegre, Digestive Diseases Surgical Unit – Porto Alegre (RS), Brazil; 2 University Hospital of Porto Alegre, General Surgery Unit – Porto Alegre (RS), Brazil.

**Keywords:** Hernia, Abdominal Wall, Hernia, Ventral, Hérnia, Parede Abdominal, Hérnia Ventral

## Abstract

**BACKGROUND:**

The development of an incisional hernia is a common complication following laparotomy. It also has an important economic impact on healthcare systems and social security budget. The mesh reinforcement of the abdominal wall was an important advancement to increase the success of the repairs and reduce its long-term recurrence. The two most common locations for mesh placement in ventral hernia repairs include the premuscular (onlay technique) and retromuscular planes (sublay technique). However, until now, there is no consensus in the literature about the ideal location of the mesh.

**AIM:**

The aim of this study was to compare the two most common incisional hernia repair techniques (onlay and sublay) with regard to the complication rate within the first 30 days of postoperative care.

**METHOD:**

This study analyzes 115 patients who underwent either onlay or sublay incisional hernia repairs and evaluates the 30-day postoperative surgical site occurrences and hernia recurrence for each technique.

**RESULTS:**

We found no difference in the results between the groups, except in seroma formation, which was higher in patients submitted to the sublay technique, probably due to the lower rate of drain placement in this group.

**CONCLUSION:**

Both techniques of mesh placement seem to be adequate in the repair of incisional hernias, with no major difference in surgical site occurrences.

## RESUMO

**RACIONAL:**

O desenvolvimento de hérnia incisional é uma complicação comum após laparotomias. Também tem um impacto econômico importante nos sistemas de saúde e no orçamento da previdência social. O reforço com tela da parede abdominal foi um avanço importante para aumentar o sucesso dos reparos e ajudou a reduzir sua recorrência em longo prazo. Os dois locais mais comuns para colocação de tela em reparos de hérnia incisional incluem os planos pré-muscular (técnica *onlay*) e retromuscular (técnica *sublay*). Porém, até o momento, não há consenso na literatura sobre a localização ideal da tela.

**OBJETIVOS:**

Comparar as duas técnicas de reparo de hérnia incisional mais comuns (onlay e sublay) em relação à taxa de complicações nos primeiros 30 dias de pós-operatório.

**MÉTODO:**

Analisar 115 pacientes submetidos a reparos de hérnia incisional *onlay* ou *sublay* e avaliar, como desfecho, as ocorrências de sítio cirúrgico no pós-operatório de trinta dias e a recorrência precoce para cada técnica.

**RESULTADOS:**

Não encontramos diferença nos resultados entre os grupos, exceto na formação de seroma, que foi maior nos pacientes submetidos à técnica de *sublay*, provavelmente pela menor taxa de colocação de dreno neste grupo.

**CONCLUSÃO:**

Assim, ambas as técnicas de colocação de tela parecem ser adequadas no reparo de hérnias incisionais, sem grande diferença nos desfechos precoces, relacionados a ao sítio cirúrgico.

## INTRODUCTION

Incisional hernia (IH) is a common complication after an open abdominal surgery, with a reported incidence of 10–20%^12,18,21^. Around 20,000 incisional hernioplasty procedures are performed annually in Brazil’s public health system (SUS). In the United States, this number reaches 200,000 procedures annually, costing from 3,900 to 16,000 dollars per surgery, depending on whether or not it requires hospitalization^20^.

Aside from the functional, aesthetic, and psychological impairment, IHs also have a large economic impact. In Brazil, they are one of the main causes of absence from work. In 2018, approximately 1% of 2,271,033 benefits granted by the Brazilian social security program were related to incisional ventral hernia (about 19,000 benefits), which represented an impact of almost 5 million dollars on the social security budget that year^9^. For comparison, it is estimated that the total cost of IH repairs in the United States is around $3.2 billion annually^5^.

Basta et al.^2^ conducted a scientific analysis of approximately 30,000 abdominal surgeries performed between 2005 and 2016, including intra-abdominal, urological, and gynecological procedures. It was identified an IH incidence of about 3.8% at an average follow-up of 57.9 months. The procedures most involved in the development of IH were colorectal (7.7%), vascular (5.2%), bariatric (4.8%), and organ transplant surgery (4.5%).

Many risk factors for IH have been identified, such as obesity, smoking, chronic obstructive pulmonary disease (COPD), previous abdominal surgery, surgical site infection (SSI), and diabetes^2,17^. Some authors have dedicated themselves to create risk stratification models to identify these high-risk patients and propose strategies to decrease their incidence^22^. It is well established that the use of mesh drastically increases the success rate of IH repairs and decreases its occurrence when used prophylactically in laparotomies^1,3,6,7,19^.

There are several approaches for mesh placement, but the most used are the onlay and the sublay (retromuscular) repairs^8^. Over the years, several studies have compared the two techniques in order to identify which has the best outcomes related to surgical site complications and recurrence. Although some have demonstrated that the sublay technique may have lower surgical site occurrences (SSOs)^10,23^, there is no consensus on which one is best to perform.

This study aimed to compare the two most common IH repair techniques (onlay and sublay) with regard to the complication rate within the first 30 days of postoperative care. Similarly, it also aimed to assess the epidemiological profile of the patients undergoing incisional hernioplasty in our institution.

## METHODS

The patients submitted to IH repairs from January 2019 to November 2020, in the University Hospital of Porto Alegre (HCPA), Brazil, funded by the National Public Health System (SUS), were retrospectively analyzed. The procedures were performed in a teaching hospital by surgeons with different levels of expertise. The institutional Ethics Committee approved the study and waived written informed consent (number 3094539).

All the incisional hernioplasty with mesh placement performed at the institution in the informed period was analyzed. Data were extracted from medical records, using an electronic standard form. We collected data about the preoperative conditions (comorbidities, imaging examinations, previous surgery, body mass index [BMI], hernia parameters, and surgery indication), intraoperative periods (surgical technique, suture thread type, and mesh parameters), and 30 days postoperative complications.

**Figure 1:**
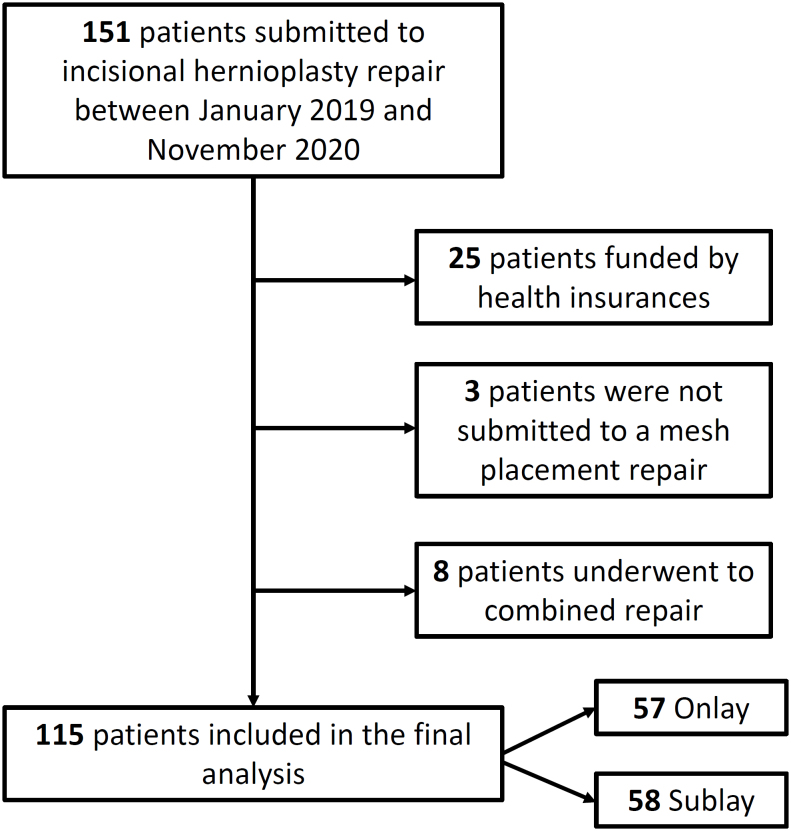
Flowchart of the article selection process.

Patients were excluded from the study if one of the following conditions was presented: age less than 14 years, repair with no mesh placement, health insurance financing, and combined surgical technique (onlay and sublay mesh replacement). Of the 151 initially identified patients, 36 were excluded from the analysis (25 patients were funded by health insurances, with no postoperative adequate follow-up data; 3 patients were not submitted to a mesh repair hernioplasty; and 8 patients were submitted to a combined repair) (Figure 1).

The European Hernia Society (EHS) classification was employed to locate the hernial defect in the abdominal wall. Midline IHs were classified into five zones: M1 subxiphoidal, M2 epigastric, M3 umbilical, M4 infraumbilical, and M5 suprapubic. Lateral IHs were classified into four zones: L1 subcostal, L2 flank, L3 iliac, and L4 lumbar. Midline hernial defects that affected the entire incision, extending over two or more zones, were classified into a separate category.

### Statistical Analysis

Data were presented as mean and standard deviation or median and interquartile range (IQR) (continuous data) or as count and proportion (categorical and ordinal data). Continuous variables with normal distribution were analyzed with the Student’s t-test and asymmetric variables with the Mann-Whitney U test. Categorical variables were compared using the chi-square test. After the univariate screening, we used multivariable linear regression to adjust for clinically and statistically significant covariates. All statistical analyses were performed with IBM^®^ SPSS^®^ *Statistics 23.0* (SPSS Inc., Chicago, IL, USA). All tests were two-sided and p-value of <0.05 was considered statistically significant.

## RESULTS

### Patients

Of the 115 selected patients, 57 underwent onlay and 58 sublay mesh placement. The patients were initially compared based on sociodemographic and comorbidity profiles (Table 1). The following comorbidities were evaluated: obesity (BMI>30), hypertension, diabetes, current neoplasia, HIV infection, immunosuppression (use of corticosteroids and immunobiologicals), coagulopathy or use of anticoagulants, chronic kidney disease, inflammatory bowel disease, and COPD. There was no statistically significant difference between the groups with regard to comorbidities. The average age between the groups was also similar, with the majority of patients being female in both groups. As part of our preoperative routine, we usually adopt a BMI<33 as a cutoff point for surgical indication, regardless of the type of technique employed. In the onlay group (OG), 47 (82.5%) patients had a BMI<33, while in the sublay group (SG), 44 (77.2%) patients with this characteristic were found; there was no statistically significant difference between the groups (p=0.484).

**Table 1:** Preoperative characteristics.

	Onlay group (57)	Sublay group (58)	p-value
Age, years	61.0±12.0	58.7±12.8	0.328
Male	16 (28)	22 (38)	0.261
BMI, kg/m2	29.7±5.8	30.3±4.4	0.608
Obesity (BMI≥30)	24 (42)	31 (54)	0.190
Smoking	9 (16)	21 (36)	0.013
Diabetes	15 (26)	14 (24)	0.788
Cancer	9 (16)	7 (12)	0.564
Immunosuppression	11 (19)	5 (9)	0.106
Chronic renal disease	1 (2)	3 (5)	0.317
Any other comorbidity	41 (72)	43 (74)	0.790

BMI: body mass index. Data are presented as mean±SD or n (%).

### Hernia Characteristics

The hernial defects of the OG had a larger total area compared to the SG (Table 2). The length and width of the defects were measured during the surgery. In case of missing measurement records in the surgical description, we used imaging tests to assess the size of the defects (mostly CT scan). Similarly, it was observed that recurrent hernias were also more frequent in the OG (14 vs. 5%, p=0.009). When analyzing these recurrent cases, we observed that 36% of patients had previously been submitted to surgery with no mesh placement, 50% underwent the onlay technique, and 14% underwent the sublay technique. In contrast, all patients of the SG with recurrent IHs had previously been submitted to the onlay repair technique.

**Table 2:** Hernia characteristics.

Hernia length, *cm*		9.6±11.9	9.4±6.2	0.889
Hernia width, *cm*		6.8±6.8	6.1±3.4	0.465
Hernia area, *cm* ^2^		79.8±11.8	65.4±61.1	0.013
Previous hernioplasty		14 (25)	4 (7)	0.009
Location (EHS)				
	M1 (subxiphoidal)	0	1 (2)	0.119
	M2 (epigastric)	15 (26)	27 (47)	
	M3 (umbilical)	12 (21)	5 (9)	
	M4 (infraumbilical)	6 (11)	6 (10)	
	M5 (suprapubic)	1 (2)	2 (3)	
	L1 (subcostal)	8 (14)	1 (2)	
	L2 (flank)	7 (12)	7 (12)	
	L3 (iliac)	2 (4)	2 (3)	
	L4 (lumbar)	0	1 (2)	
	Entire incision	6 (11)	6 (10)	

EHS: European Hernia Society. Data are presented as mean±SD or n (%).

The types of procedures most involved in the development of IH in the SG were exploratory laparotomy (24.1%), colectomy (19%), and bariatric surgery (8.6%). In OG, we obtained a slightly different profile, and the most common surgeries related to IHs were colectomy (19.3%), exploratory laparotomy (17.5%), and open cholecystectomy (9%).

When analyzing the position of the abdominal wall defects according to the classification of the EHS, we observed that in the OG, epigastric hernias predominated (26%), followed by umbilical hernias (21%) and subcostal hernias (14%). In the SG, epigastric defects also predominated (47%), but it was followed by flanks (12%) and infraumbilical (10%) defects. Despite this difference was not statically significant, we believe it was due to different types of surgeries that originated the IHs in both groups.

### Surgical Techniques

All surgeries, regardless of the technique, were performed using a polypropylene mesh. We also observed that in the OG, all surgeries involved mesh fixation, while in the SG, 16% of the cases were performed without any type of mesh fixation (p=0.002). Polypropylene (58%) was the most used type of suture thread to fixate the mesh, followed by polydioxanone (23%) and polyglactin (19%). Most surgeries were performed using the open technique (95%).

There was a higher rate of drain placement in the OG, probably due to the need for greater dissection of the subcutaneous tissue, which also could lead to other postoperative complications, such as seroma formation. In all cases where drainage was performed, it was used a suction drain (e.g., Portovac^®^).

Although the abdominal wall defects in the patients of the OG were larger (Table 2), the average size of the mesh used was not. In fact, the mesh length was greater in the SG (Table 3). A total of eight patients of the OG group and three patients of the SG underwent emergency surgery, all due to incarcerated hernia, with no statistical difference (p=0.106).

**Table 3:** Surgical parameters.

		Onlay group (57)	Sublay group (58)	p-value
Absence of mesh fixing		0	9 (16)	0.002
Type of suture thread				
	Polypropylene	34 (60)	28 (57)	0.318
	Polyglactin	8 (14)	12 (25)	
	Polydioxanone	15 (26)	9 (18)	
Drain		41 (72)	17 (30)	<0.001
Type of repair				
	Open	57 (100)	52 (90)	0.013
	Videolaparoscopic	0	6 (10)	
Mesh length, *cm*		16.2±8.2	19.8±6.8	0.021
Mesh width, *cm*		14.3±7.8	16.4±6.1	0.156
Mesh area, *cm* ^2^		287.9±290.1	350.8±210.5	0.066
ASA classification				
	1	7 (12)	2 (3)	0.208
	2	36 (63)	43 (74)	
	3	14 (25)	12 (21)	
	4	0	1 (2)	

ASA: American Society of Anesthesiology. Data are presented as mean±SD or n (%).

### Postoperative data

The postoperative data are shown in Table 4. All postoperative outcomes were evaluated within the first 30 days after surgery, either during hospitalization or outpatient visits. We assessed SSOs, which included patients who presented at least one of the following surgical wound complications: infection, seroma, fistula, and dehiscence. Similarly, each of these complications was individually identified and compared between the groups.

**Table 4:** 30-Day postoperative data.

		Onlay group (57)	Sublay group (58)	p-value
Surgical site infection		9 (16)	8 (14)	0.762
Seroma		12 (21)	23 (40)	0.030
Fistula		0	1 (2)	0.319
Wound dehiscence		9 (16)	10 (18)	0.834
Hernia recurrence		2 (4)	2 (4)	0.986
Death		2 (4)	1 (2)	0.548
Surgical site occurrence		24 (42)	28 (48)	0.506
Clavien-Dindo				
	Grade 1	14 (58)	17 (61)	0.661
	Grade 2	6 (25)	7 (25)	
	Grade 3	2 (8)	2 (7)	
	Grade 4	0	1 (4)	
	Grade 5	2 (8)	1 (4)	

Data are presented as mean±SD or n (%).

A total of 52 (45%) patients had some type of postoperative complications. The most common complication was seroma formation (30%), followed by skin dehiscence (17%) and SSI (15%). Complication rates, analyzed either individually or together (SSO), were similar between groups, with the exception of seroma formation. We observed a higher incidence of seroma in the SG compared with the OG (40 vs. 21%, p=0.030). Considering the higher rate of drain placement in the OG, we performed a linear regression to control this variable and found that the placement of drains was related to a lower incidence of seroma formation. Postoperative complications were also evaluated using the Clavien-Dindo scale, and no statistically significant differences were found between the groups (Table 4).

## DISCUSSION

The groups in our study had no statistically significant difference in terms of preoperative characteristics, showing that the comparison between the two is feasible. Most of the patients were female, with an average age of 60 years and a BMI of 30 kg/m^2^. There are some well-known risk factors associated with IHs and recurrence. Some of them include obesity, smoking, malnutrition, old age, immunosuppression, and connective tissue disorders^4,11^. Therefore, it is also important to note that the groups in our studies were similar with regard to these risk factors. We found a recurrence rate of 4% in both groups, with no statistical difference between them. Similar results were found by Demetrashvili et al.^10^, who compared the onlay and retromuscular techniques in 180 hernia cases and showed that there was no difference in recurrence.

Wound complications are a common problem in IH repair, regardless of the technique. Some studies have shown that the development of these complications occurs more frequently after onlay repair compared to the retromuscular method^24^, although others do not^14,25^. Seroma formation is one of the common complications, with an incidence of 30–50% after open mesh repair. The exact pathophysiology of seroma formation is unknown^13^. Some authors justify that both seroma and infection are more frequent after the onlay technique due to greater dissection of the subcutaneous tissue and its contact with the mesh^15^.

Recent meta-analyses comparing retromuscular and onlay repair techniques did not show a difference in seroma development, but fewer cases of wound infection were found in the retromuscular group. The higher incidence of wound infection after onlay hernia repair might be explained by the superficial location of the mesh and the facilitation of bacterial colonization in the area^10,15^.

In contrast, Demetrashvili et al.^10^ showed a lower rate of wound complications when comparing retromuscular hernia repair (22.1%) with onlay repair (50.0%) (p<0.001). The incidence of postoperative seroma was also higher in the OG (p<0.0013). There was no difference in the frequency of wound infection and hematoma between the groups.

Ibrahim et al.^13^ conducted a systematic review to answer the following question: “Among the onlay and sublay techniques, which one offers the lowest seroma rate?” Of the 64 articles evaluated, after the exclusion criteria, a total of 6 articles (2 randomized controlled trials, 1 prospective study, and 3 retrospective studies) were chosen to provide the best evidence to answer the question. Two studies in this review did not suggest any difference in the seroma rate between onlay and sublay hernia repair. In contrast, the rest of the four studies showed a lower rate of seroma in the SG patients compared to the OG.

Our results suggest a different trend. The number of surgical site complications (SSO) did not show a difference between OG and SG. However, when individual analysis was conducted, we observed that the retromuscular group had a higher incidence of seroma compared to the OG (40 vs. 21%, p<0.030). There was no difference regarding other surgical site complications. In an attempt to explain the difference in seroma formation, we could observe that the OG had a higher rate of drain placement compared to the retromuscular group (72 vs. 30%, p<0.001). The result did not change even after controlling this variable with covariance analysis.

In contrast to our study, Westphalen et al.^26^ allocated 42 individuals with large IHs who underwent onlay mesh repair in two groups. In group 1, suction drains were placed in the subcutaneous tissue, while in group 2, there was only subcutaneous suture without drainage. Participants underwent clinical and ultrasound evaluation to detect seroma and surgical wound infection three times after surgery. They concluded that there was no statistical difference in seroma formation or wound infection frequency between groups and that drain placement does not minimize the rate of surgical site complications. Another retrospective study performed by Hodgson et al.^16^ evaluated the incidence of postoperative complications after drain placement in various types of hernia repairs. They also found that drainage did not decrease the incidence of seroma formation but only increased the time of hospitalization.

## CONCLUSION

The increased incidence of IHs has become a global trouble. Despite the advancement of surgical techniques in recent years, some aspects are still under debate. Thus, both onlay and sublay have similar results, but routine drainage can decrease the rate of seroma formation.
